# The effect of eight-week core stability training program on the dynamic balance in young elite footballers

**DOI:** 10.1186/1748-7161-8-S1-P20

**Published:** 2013-06-03

**Authors:** J Abdi, H Sadeghi

**Affiliations:** 1 Islamic Azad University, Central Tehran Branch, Teheran, Iran; 2 Orthopedic & Rehabilitation, Tehran Tarbiat Moallem University, Teheran, Iran

## Background

Core stability is the central motor control and muscular capacity of the lumbar-pelvic-thigh to maintain stability of this region against various postural and external forces[1]. Studies, have been shown different roles of core stability to improve performance; Lewarchik (2003) tend to stabilize the core in footballers not observed significant using the stability exercise program and plyometric[2]. Kahle(2009) have been shown improved postural control after six weeks core stability training program in healthy and young adults[3]. Accordingly, there are different results of research, and important training on postural stability in the football skills.

## Aim

The purpose of this study was the effect of eight-week core stability training program on the dynamic balance in young elite footballers.

## Methods

Statistical 15 football players, 19 to 24 years in FC Tehran Damash with mean age 21.10 ± 1.25 years, height 179.53 ± 6.83 cm, weight 71. 80 ± 7.42 kg, body mass index 22.23 ± 1.27 kg/m2 and a maximum vertical jump was 51.66 ± 5.58 cm. performance eight-week core stability training program with three sessions a week for 30 minutes was run. Control of dynamic balance on the force plate, at the dominant and non dominant leg, through jump-landing with a 50% vertical jump maximum, in pre and post test was evaluated by dynamic postural stability index. The data analysis by paired t-test, one-way analysis of variance (ANOVA) and Tukey post hoc test at significance level 0 / 05 was used.

## Results

Results findings a high level significantly in progress dynamic balance in the medial-lateral, anterior-posterior and vertical directions and dynamic postural stability of the whole proved.

## Discussion

The results of the study are dissimilar with the Lewarchik study. Possible causes of dissimilation are age of subjects and measuring. And similar with the Kahle study, possible reasons can be exercise protocol. Core muscle contraction of the member, the reaction between postural disorders of the central nervous system that prevents postural and core stability exercise program, can result in improved prediction of activity, and thereby reducing the disruption displacement and fluctuates of the center of gravity.
